# DNA Methylation—An Epigenetic Mark in Mutagen-Treated *Brachypodium distachyon* Cells

**DOI:** 10.3390/plants10071408

**Published:** 2021-07-09

**Authors:** Adrianna Wiktoria Bara, Agnieszka Braszewska, Jolanta Kwasniewska

**Affiliations:** Plant Cytogenetics and Molecular Biology Group, Faculty of Natural Sciences, University of Silesia in Katowice, Jagiellonska 28, 40-032 Katowice, Poland; adriannabara@gmail.com (A.W.B.); agnieszka.braszewska@us.edu.pl (A.B.)

**Keywords:** *Brachypodium*, DNA methylation, maleic hydrazide, micronuclei, N-nitroso-N-methylurea

## Abstract

The chromatin structure is significantly influenced by some epigenetic modifications including DNA methylation. The nuclear organization plays an essential role in the cell response to external stresses including mutagens. We present an analysis of the correlation between epigenetic modifications and the instability of the *Brachypodium distachyon* genome, which are observed as micronuclei, following maleic hydrazide (MH) and nitroso-N-methylurea (MNU) treatments. We compared the level of DNA methylation in the control (untreated) and mutagen-treated *B. distachyon* nuclei. An immunostaining method using specific antibodies against modified DNA anti-5-methylcytosine was used for the evaluation of DNA methylation in a single nucleus and micronucleus. Interestingly, we showed an alteration of DNA methylation in cells after mutagenic treatments. The results indicate that DNA methylation might be involved in the response of the *B. distachyon* genome to mutagenic treatments. This demonstrates that analyses of the epigenetic modifications should be integrated into current plant genetic toxicology in order to explain the mechanisms of DNA damage and repair in plants.

## 1. Introduction

The nuclear organization plays a crucial role in the cell response to external stresses. The chromatin structure, including the euchromatin–heterochromatin ratio, is one of the nuclear genome features that determine its sensitivity to environmental mutagenic factors [1]. It is known that condensed chromatin can even play a protective role for transcriptionally active euchromatin [2]. The chromatin structure represents a highly dynamic configuration, and it is significantly influenced by some epigenetic modifications, including DNA methylation. This process involves the covalent addition of a methyl group to the fifth position of cytosine in the pyrimidine ring at the chemical level, which is catalyzed by the methyltransferase enzymes using S-adenosyl methionine as the methyl group donor. The spatial distribution and organization of chromatin as well as dynamic changes, including epigenetic chromatin modifications, have frequently been evaluated to study the plant cell response to environmental factors [3,4]. Stresses such as treatment with aluminum, salt, cold or drought can induce changes in the gene expression via the hypomethylation or hypermethylation of DNA [5]. An analysis of the dynamic nature of nuclear changes also seems to be a promising field in mutagenesis today. It has become clear that the nuclear architecture, including epigenetic chromatin modifications, has a broad functional role in the response of mutagen-treated cells [6].

Changes in the epigenetic modifications strongly depend on the type of mutagen, e.g., gamma rays cause significant changes in the DNA methylation level. In contrast, maleic hydrazide (MH) induces changes in the histone methylation and acetylation levels [7]. Moreover, it was suggested that the global DNA methylation in mutagen-treated cells might be involved in specific aspects of DNA repair [8]. The role of DNA methylation was proven to play a role in human carcinogenesis and cancer therapies [9]. More data on the role of the epigenetic modifications in DNA damage and repair are needed.

Many plant genotoxicity tests have been developed to investigate the cytogenetic effects of various agents that potentially induce DNA damage. Among them, the micronuclei (MN) test, which was proposed by Evans et al. [10], is widely used to analyze the genotoxic effect of a large number of chemical and physical agents [11,12,13,14,15,16]. There is also an example of using the MN test in *Brachypodium distachyon* (L.) *P. Beauv.* cells [17,18,19]. Micronuclei are defined as small, extranuclear bodies that are formed from chromosome fragments or entire chromosomes as a result of the chromosome breakage, kinetochore damage or disturbances of the cell cycle. The unrepaired DNA double-strand breaks that result from the misrepair of these strand breaks may lead to MN formation [20]. The molecular cytogenetic approaches, especially fluorescence in situ hybridization (FISH), are very useful in detecting micronuclei and identifying specific chromosomes or chromosome fragments in the micronuclei to determine their origin [21,22,23,24], especially in species with a small genome [17,18,19]. However, an understanding of the mechanisms of MN formation is still incomplete. *Brachypodium distachyon* is a widely accepted model grass with numerous favorable features and resources, such as a small, simple and fully sequenced nuclear genome, small plant stature and rapid life cycle [25,26]. *B. distachyon* is a therophytic species, widespread in Europe where it characterizes annual meadows protected by Habitat Directive 92/43 EEC as a priority habitat called a Pseudo-steppe with grasses and annuals of the Thero-Brachypodietea (6620* code) [27] plant community that forms a mosaic with many other types of perennial vegetation [28]. Vogel and Hill [29] demonstrated that *B. distachyon* is a promising plant for mutagenesis purposes. Protocols have been established for the mutagenesis of *B. distachyon* with sodium azide [30,31], fast neutron radiation [32] and gamma radiation [33]. Our previous studies have shown a positive response of *B. distachyon* to mutagenic treatments using MH and x-rays [17,18,19]. *B. distachyon* was proven to be well suited for analyzing the plant nuclear genome stability. There are cyto-molecular resources, including the FISH-based and chromosome-specific approaches such as chromosome barcoding and chromosome painting, that enable a detailed insight to be obtained into the micronuclei structure that is induced by mutagens. The involvement of specific DNA sequences in micronuclei formation has shown that the distribution of the DNA break points is not random. Chromosome size, gene density and other aspects of the chromatin organization are the factors that influence the preferential origin of the MN from specific chromosomes in animals and humans [34,35,36,37].

At present, the methodological possibilities enable some essential questions regarding the correlation between chromatin structure, epigenetic modifications and the instability of the plant genome in mutagenesis to be addressed. In this study, we analyzed the level of DNA methylation, which is a well-known heterochromatin marker in the nuclei and micronuclei in *B. distachyon* after chemical mutagenesis. Mutagens that are characterized by different mechanisms of action: maleic hydrazide (MH), which is a clastogenic agent that can lead to chromosome breaks and also cause spindle fiber defects [38], and nitroso-N-methyl-urea (MNU), which is an alkylating agent that mainly induces gene mutations but also leads to chromosomal aberrations, were used to induce micronuclei in *B. distachyon* root meristematic cells [19]. The mutagens used in this study act in the different cell cycle phases: MH in the S phase and MNU in the G2 phase [39]. The aim of this study was to analyze and compare the level of DNA methylation in the control (untreated) and mutagen-treated *B. distachyon* nuclei. An immunostaining method using specific antibodies against modified DNA anti-5-methylcytosine was used to determine the presence and level of DNA methylation that were eliminated from the nucleus as micronuclei.

## 2. Results

### 2.1. Micronuclei in the B. distachyon Cells

**Figure 1:**
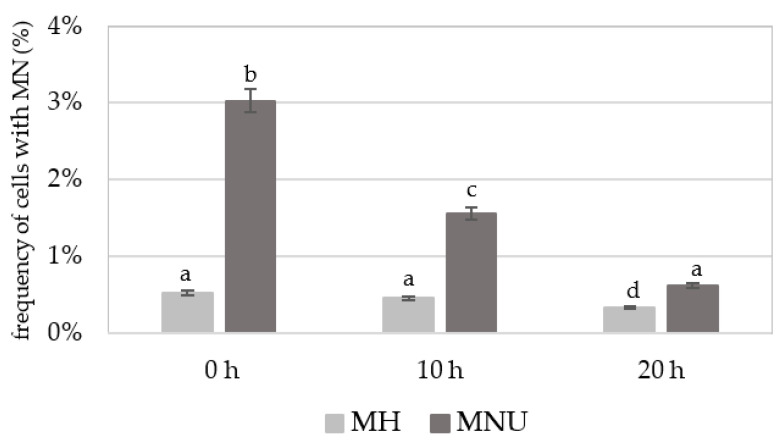
Frequency of *B. distachyon* cells with micronuclei (MN) after MH and MNU treatment. No micronuclei were observed in the control cells. ANOVA followed by Tukey HSD test, *p* < 0.05; mean ± SD, statistically significant differences are indicated by different letters.

Before detecting 5 mC, the slides with the nuclei were stained with DAPI. The frequencies of *B. distachyon* root meristematic cells that formed micronuclei (control and treated) were estimated. The number of micronuclei in a single cell was not higher than one. The frequency of micronuclei after treatment with MH or MNU varied from 0.53% to 3.03% (Figure 1). The highest frequency of cells with micronuclei was observed at 0 h after treatment after which it decreased with the posttreatment time. However, at 20 h after treatment with MH or MNU, cells with micronuclei were still observed. No MN were observed in the control cells.

### 2.2. The Presence of 5 mC Signals in the Control and Mutagen-Treated B. distachyon Cells

**Figure 2:**
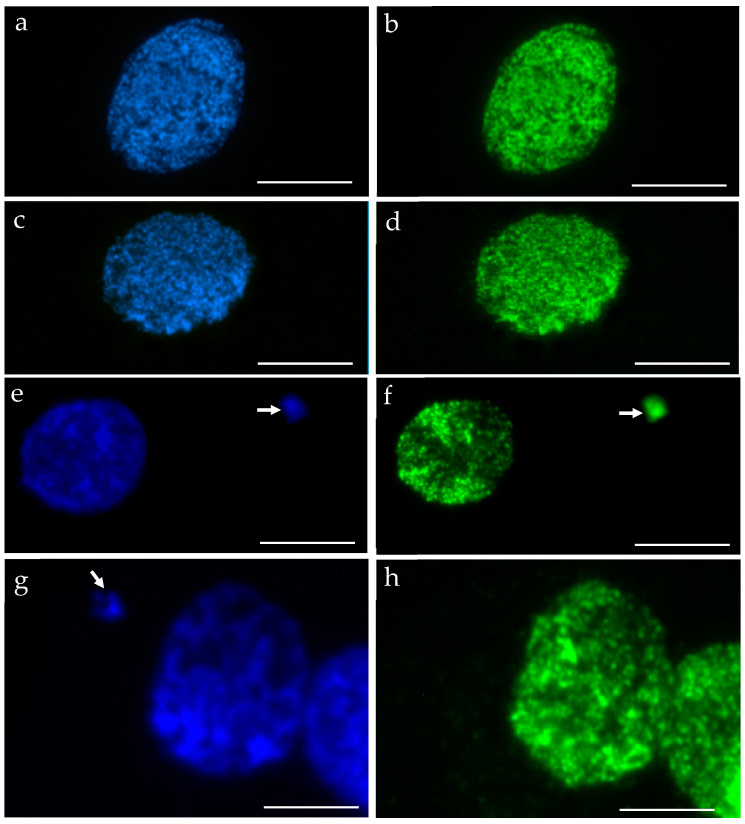
*B. distachyon* interphase nuclei: DAPI stained, blue (**a**,**c**,**e**,**g**) that were used for the immunocytochemical detection of 5 mC, green (**b**,**d**,**f**,**h**): (**a**,**b**) control, (**c**–**h**) treated with MH. Nuclei without micronuclei (**a**–**d**) and with micronuclei (**e**–**h**) were observed. The arrows indicate the micronuclei. The 5 mC signals were always present in the nuclei. Two different kinds of micronuclei were distinguished: those with 5 mC signals (**e**,**f**) and those without signals (**g**,**h**). Scale bars = 10 µm.

**Figure 3:**
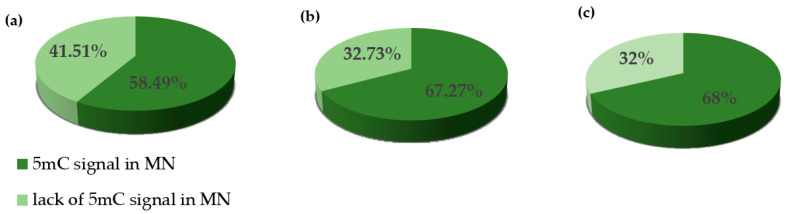
The frequencies of micronuclei (MN) with and without 5 mC signals after **MH treatment** at different posttreatment times: (**a**) 0 h, (**b**) 10 h, (**c**) 20 h.

**Figure 4:**
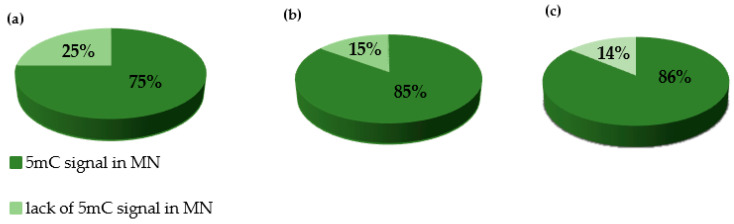
The frequencies of micronuclei (MN) with and without 5 mC signals after **MNU treatment** at different posttreatments times: (**a**) 0 h, (**b**) 10 h, (**c**) 20 h.

We performed the procedure of the immunocytochemical detection of **5**-methylcytosine (5 mC) with the secondary antibody conjugated with Alexa Fluor 488 and without the primary antibody. No unspecific binding of the secondary antibody to these samples was detected. The 5 mC signals were always observed in the nuclei in both the control and treated nuclei, whereas the micronuclei that were induced by MH or MNU were either labeled by 5 mC or not. The examples of control nuclei and nuclei with and without micronuclei after MH treatment are shown in Figure 2. We analyzed the frequencies of the micronuclei (MN) with and without 5 mC signals after MH (Figure 3) and MNU treatments (Figure 4) in the *B. distachyon* cells, followed by different posttreatment times: 0 h, 10 h, 20 h. The frequency of MN with 5 mC signals differed in the MH- and MNU-treated cells. It was higher after the MNU treatment and ranged from 75% to 86%, whereas after MH treatment, it was 58.49–68%. The use of posttreatment times after mutagenic treatment with MH and MNU caused an increase in the frequency of MN with 5 mC signals. The frequency of the MN with 5 mC signals increased during the subsequent postincubation times by up to 10% after both MH and MNU treatments.

### 2.3. The Level of 5 mC in the Control and Mutagen-Treated B. distachyon Cells

**Figure 5:**
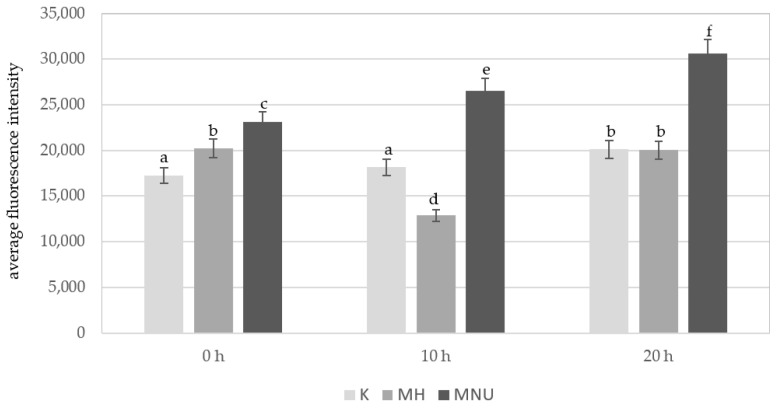
Comparison of the average fluorescence intensity of Alexa 488 that was used to detect 5 mC in the *B. distachyon* nuclei, without MN: in the control and MH- or MNU-treated cells at different posttreatment times. Fluorescence of Alexa 488 was measured in relative units. ANOVA followed by Tukey HSD test, *p* < 0.05; mean ± SD, statistically significant differences are indicated by different letters.

An analysis of the average fluorescence intensity of Alexa 488 in the *B. distachyon* nuclei without micronuclei revealed differences between the control and the MH- and MNU-treated cells (Figure 5). At 0 h, the lowest fluorescence intensity of Alexa 488 was observed in the control cells, this significantly increased after the MH treatment and especially after the MNU treatment. The highest fluorescence intensity of Alexa 488 was observed after the MNU treatment. In the control cells, the 5 mC level was very similar at 0 h and 10 h of the posttreatment time and significantly increased at 20 h. After the treatment with MH, there was a significant decrease in the fluorescence intensity at 10 h of posttreatment, and at 20 h it increased to the same value as at 0 h. The effect of the posttreatment time on the 5 mC level was also observed after the treatment with MNU. The intensity of Alexa 488 increased by 13% at 10 h and 25% at 20 h compared to 0 h.

**Figure 6:**
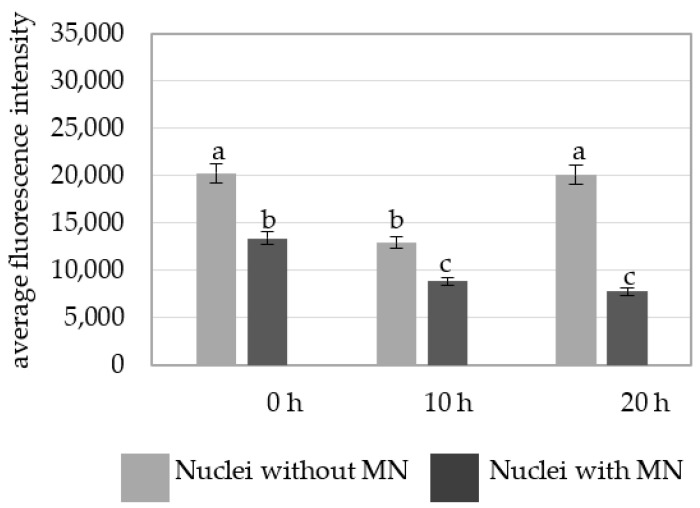
A comparison of the average fluorescence intensity of Alexa 488 that was used to detect 5 mC in the *B. distachyon* nuclei with and without micronuclei in the MH-treated cells at different posttreatments times. Fluorescence of Alexa 488 was measured in relative units. ANOVA followed by Tukey HSD test, *p* < 0.05; mean ± SD, statistically significant differences are indicated by different letters.

A comparison of the average fluorescence intensity of Alexa 488 in the nuclei with and without micronuclei in MH-treated cells at different posttreatment times showed that the nuclei without micronuclei were characterized by a higher fluorescence intensity than in the parental nuclei for the micronuclei (Figure 6).

**Figure 7:**
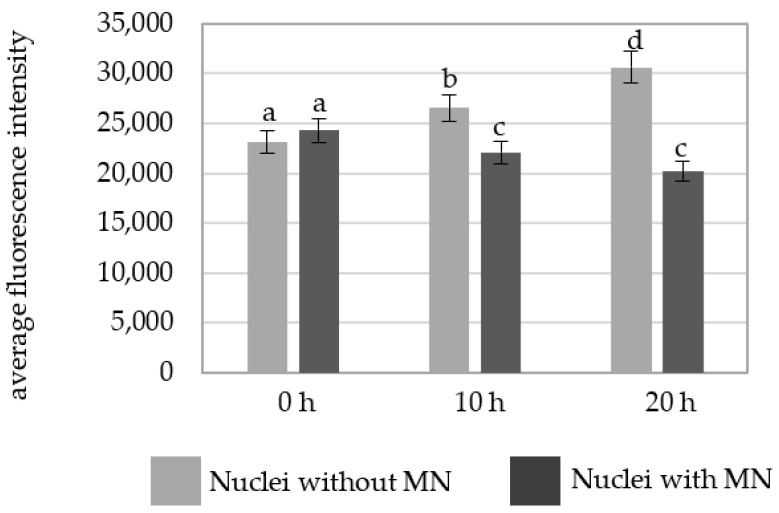
A comparison of the average fluorescence intensity of Alexa 488 that was used to detect 5 mC in the *B. distachyon* nuclei with and nuclei without micronuclei in the MNU-treated cells at different posttreatment times. Fluorescence of Alexa 488 was measured in relative units. ANOVA followed by Tukey HSD test, *p* < 0.05; mean ± SD, statistically significant differences are indicated by different letters.

The intensity of Alexa 488 fluorescence in the MNU-treated cells was also higher in the cells without micronuclei at 10 h and 20 h (Figure 7).

**Figure 8:**
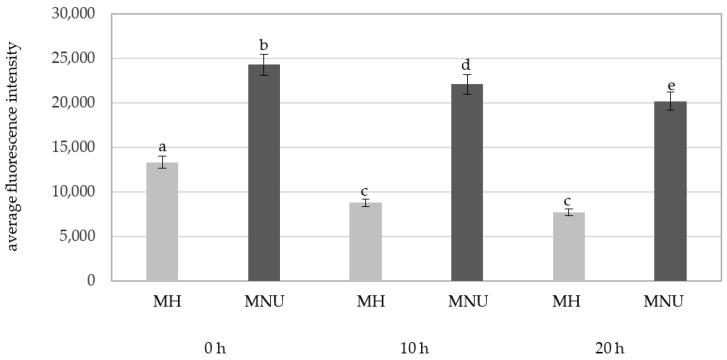
A comparison of the average fluorescence intensity of Alexa 488 that was used to detect 5mC in the *B. distachyon* parental nuclei with micronuclei in MH- and MNU-treated cells at different posttreatments times. Fluorescence of Alexa 488 was measured in relative units. ANOVA followed by Tukey HSD test, *p* < 0.05; mean ± SD, statistically significant differences are indicated by different letters.

There were significant differences in the fluorescence intensity of Alexa 488 in the parental nuclei for micronuclei between the MH and MNU treatments (Figure 8). The MNU-treated parental nuclei were characterized by a higher fluorescence intensity than the MH-treated parental nuclei.

**Figure 9:**
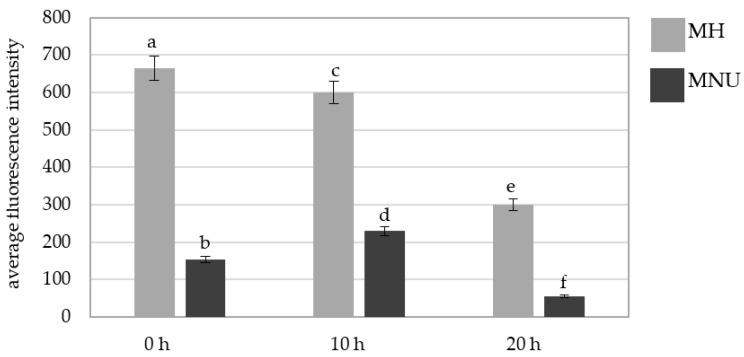
A comparison of the average fluorescence intensity of Alexa 488 that was used to detect 5 mC in the *B. distachyon* micronuclei in the MH- or MNU-treated cells at different posttreatment times. Fluorescence of Alexa 488 was measured in relative units. ANOVA followed by Tukey HSD test, *p* < 0.05; mean ± SD, statistically significant differences are indicated by different letters.

We also analyzed the fluorescence intensity of Alexa 488 in the micronuclei that were induced by MH and MNU (Figure 9). The Alexa 488 fluorescence intensity differed for the MH- and MNU-induced micronuclei; it was significantly higher in the micronuclei that formed as a result of the MH treatment at all of the posttreatment times. A decrease in the Alexa 488 fluorescence intensity in MH- and MNU-induced micronuclei at 20 h compared with 0 h was observed with the posttreatment time by 55% after the MH treatment and 75% after the MNU treatment.

## 3. Discussion

Environmental epigenetics is a rapidly expanding area of research. Different environmental chemicals that can modify epigenetic marks are known in humans and animals [40]. Hypermethylation is the default epigenetic state and assists in maintaining genome integrity. Several genetic studies have indicated that global DNA hypomethylation is associated with increased genome instability such as changes in ploidy and chromosomal abnormalities [41]. The connection between DNA hypomethylation and genome instability is especially well documented in the context of cancer [42]. While there are numerous molecular studies on the role of various epigenetic modifications in DNA damage and repair in plants [43,44,45,46,47,48,49], there is still a lack of analyses of these changes in single nuclei in respect to chromosome aberrations.

We analyzed the DNA methylation in *B. distachyon* single nuclei and micronuclei after mutagenic treatments with two chemical mutagens—MH and MNU. The evaluation of the micronuclei (MN) formation after mutagenic treatment, which was used in this study, is commonly used to measure a compound’s clastogenic effect [11]. DNA damage, together with DNA repair, can be evaluated using the MN test, because the double-strand breaks (DSBs) that are caused by mutagens, if not repaired, can lead to micronuclei formation. We showed that MH and MNU induced micronuclei in the *B. distachyon* root meristematic cells. The clastogenic effect of MH in *B. distachyon* was previously reported by our group [17,18,19], whereas MNU was proven to induce micronuclei in this species for the first time. Whether micronuclei can be re-engulfed by the cell nucleus [50] and whether the micronuclear content can be degraded independently of further cell division are still unknowns [51]. Most probably, micronuclei are considered to be genetic material that is lost from a cell. A decrease in the frequency of micronuclei with the posttreatment times was observed after the MH and MNU treatments, which proves that the micronuclei are eliminated from the cells [12]. This decrease was accompanied by an increase in the number of methylated MN, which suggests that DNA methylation may be correlated to the elimination of chromatin from the nucleus as MN.

We analyzed the presence and the level of **5**-methylcytosine using the fluorescence intensity of Alexa 488 in *B. distachyon* nuclei and micronuclei after mutagenic treatment. To date, different molecular approaches have been developed to map genome-wide DNA methylation. The bisulfite sequencing approach would be helpful to map single-cell genome-wide DNA methylation in animals [52] and plants [53,54]; nevertheless, this does not enable the analysis of the DNA methylation in relation to chromosomes aberrations. The bisulfite sequencing approach will technically not be feasible in single nuclei and micronuclei due to the extremely small size of Brachypodium nuclei, and especially micronuclei, which prevents their isolation. The cytogenetic approach is time-consuming; however, it enables researchers to perform an in situ analyses within a single micronucleus together with its parental nucleus. Unfortunately, this method is much more time-consuming than the molecular methods. Flow cytometry offers an automated high-throughput platform that is reproducible; however, it needs to be standardized for each plant species and the fluorochrome that is to be used and does not provide data on the in situ localization of DNA methylation. A similar methodological approach using manual scoring with a microscope and the appropriate software for analyzing DNA methylation was previously used in human cells [55].

Our data on the level of DNA methylation in nuclei and micronuclei suggest the possible involvement of epigenetic mechanisms in the (in)stability of the *B. distachyon* genome when subjected to mutagens. 5 mC fluorescence intensity showed the level of DNA methylation slightly changed in the control during the applied posttreatments time, although we provided the controlled growth conditions. Such changes, which take place across time during root development, have been observed previously [56]. The fluctuations of fluorescence intensity corresponding to 5 mC across time in mutagen-treated cells was completely different. Therefore, we can cautiously conclude that the fluctuation of fluorescence intensity after mutagen treatment is not only related to the level of 5 mC in the control but also to mutagen action and specific DNA repair processes. This is also supported by the fact that different types of fluctuation were shown for MH and MNU treatment, which were characterized by different mechanisms of action. Unexpectedly, the average fluorescence intensity of Alexa 488 that was analyzed in the nuclei that did not form micronuclei increased after MNU treatment compared to the control, and it also increased with posttreatment times, thereby indicating that DNA hypermethylation processes occurred. The different response that was observed after the MH treatment could have resulted from a different mechanism of action of the applied mutagens. Simultaneously, the significantly lower level of 5 mC in the parental nuclei for MN compared with the nuclei without MN after both the MH and MNU treatments, as well as the decreasing level of 5 mC in the micronuclei during the posttreatments, could indicate that the formation and elimination of MN was somehow correlated epigenetically via the loss of DNA methylation. The decreased level of 5 mC with the posttreatment times indicates that there are active processes of DNA demethylation in the MN. This scenario is possible if DNA replication takes place in the MN [57], which can be proven by using the “click” reaction with **5**-ethynyl-**2**′-deoxyuridine (EdU) [58]. However, it is also possible that the decrease in the level of 5 mC in the parental nuclei could be the result of the loss of DNA into the MN and as the consequence of DNA elimination from nuclei. Surprisingly, the number of micronuclei with 5 mC signals increased with postincubation times. The hypomethylation of heterochromatin in the pericentromeric region is associated with chromatin decondensation, which leads to improper chromosome segregation and exclusion into the MN in humans [59]. The presence of Alexa 488 signals in the micronuclei indicates the hypermethylated DNA localization. In plants, DNA methylation is restricted to specific genomic regions, e.g., in Arabidopsis, and most of the methylated DNA is composed of local tandem or inverted repeats, transposons and other dispersed repeats around the centromeres and in the euchromatin [60]. A similar localization in the pericentromeric regions has been shown for *B. distachyon* [61]. Thus, because most of the micronuclei contain the signals of Alexa 488, they probably originated from the chromosome fragment(s) of the pericentromeric region(s), which are hypermethylated as a result of chromosome breakage or whole chromosomes as a result of kinetochore disturbances. It was previously shown that the localization of chromosome aberrations within the *B. distachyon* genome is not random [17,18,19]. On the other hand, methylation of the coding regions is common among eucaryotes and, therefore, could be a preventive mechanism for micronuclei formation [62]. According to Fenech et al. [20], the main mechanism of MN formation that originates from chromosome missegregation is the hypomethylation of the centromeric and paracentromeric regions—the satellite repeats. The epigenetic mechanisms of MN formation in animals were revealed using folate, which is a B group vitamin that is crucial for DNA methylation [63]. Our research is a rare example of the analysis of the DNA methylation in relation to the chromosome aberrations, namely micronuclei in plants. Previously similar studies were performed in humans [64]. The authors undoubtedly evidenced the contribution of epigenetic alterations to MN formation in human cells; micronuclei formation is induced epigenetically, mainly through the loss of DNA methylation.

The characteristic response of genomes, which are visible as changes in DNA methylation level for genotoxic agents, could be due to a specific mechanism of action of mutagens. MH is a well-described clastogenic mutagen that acts in the S phase, whereas MNU causes point mutations, although chromosome damage is also a characteristic for it. These specific effects regarding DNA methylation in response to two chemical mutagens need to be determined in studies with a larger number of chemical and physical compounds that are characterized by different mechanisms of action.

The number of studies on the epigenetic impact of mutagens on the plant genome and the contribution of epigenetic alterations in MN formation is limited and the molecular mechanisms of their influence remain unknown. However, some hypotheses considering DNA hypomethylation in MN formation are postulated. Hypomethylation of repeated DNA sequences, such as satellite DNA in the centromeric and pericentromeric regions of chromosomes, could be linked with chromosome instability. Specifically, hypomethylation of heterochromatin in the pericentromeric regions is associated with chromatin decondensation, which leads to improper chromosome segregation and exclusion into MN, whereas global hypomethylation is associated with more relaxed chromatin, increased gene expression, elevated DNA damage and chromosomal breaks that form MN with acentric chromosome fragments [64]. The obtained results indicated hypomethylation in nuclei that form MN and, additionally, in MN after treatment with MH. The effect of hypomethylation and MN formation was similar to other research in humans [65,66]. The current data indicate that DNA methylation is somehow involved in the genotoxicity effects in plants that are caused by MH and MNU. Thus, we proposed that this could be used as a genotoxicity endpoint together with micronuclei formation. Other epigenetic modifications, such as H2AX phosphorylation, served as an early marker for DNA damage in cancer predictivity [55]. Bleomycin and MMS led to the exclusion of the H2AX phosphorylation foci from heterochromatin into the MNs within the majority of nuclei. H2AX foci have also been largely excluded from heterochromatin after irradiation [67]. DNA methylation should be integrated into current genetic toxicology in order to explain the mechanisms of action of genetic instability.

## 4. Materials and Methods

### 4.1. Plant Material, Mutagenic Treatment and Slide Preparation

The plant material used in the study was *Brachypodium distachyon* seeds (Brachypodium, 2*n* = 10, cv. B21). The seeds were pre-soaked in water for 6 h, then sown in Petri dishes on moist filter paper and germinated at 21 °C in the dark for 72 h. The seedlings were treated with maleic hydrazide (MH, 4 mM; Sigma, CAS 123-3301) or nitroso-N-methyl-urea (MNU, 3 mM, Sigma, CAS 684-93-5) for 3 h in the dark under aeration at 21 °C. The mutagenic treatment procedure was repeated twice. The treatment conditions with MH used in the study were used in previous experiments in which the cytogenetic effects were estimated in *B. distachyon* [17,18,19]. The control seedlings were incubated under the same conditions in distilled water. After the treatment, the seedlings were washed three times in distilled water and then germinated in Petri dishes. The material was fixed in ethanol–glacial acetic acid (3:1) at three postincubation times: 0 h, 10 h and 20 h after treatment. The roots of the seedlings were used as the source of the meristems for the investigations of the micronuclei. For the nuclei and micronuclei preparations, the material was washed with a 0.01 mM sodium citrate buffer (pH 4.8) for 30 min and digested with an enzyme mixture containing 1% pectinase (*v/v*, Sigma) and 2% cellulose (*w/v*, Sigma) for 1.5 h at 37 °C. After digestion, the material was washed again with a sodium citrate buffer for 30 min. The squash preparations were prepared in a drop of 45% acetic acid. After freezing and coverslip removal, the slides were dried.

### 4.2. Immunostaining

The immunostaining step in *B. distachyon* was carried out as was previously described for barley with minor modifications [68]. The immunodetection of **5**-methylcytosine (5 mC) within nuclei and MN was performed using the primary antibody-mouse anti-**5**-methylcytosine (1:100 dilution in 1% BSA in 1 × PBS, Abcam, cat. no. ab73938), and then detected by the secondary antibody goat anti-mouse IgG antibodies (1:100 dilution in 1% BSA, Invitrogen, Molecular Probes, cat. no. A-11001), which had been conjugated with Alexa Fluor 488.

Prior to the immunodetection of 5 mC, a chemical denaturation was performed with a solution of 0.25 M sodium hydroxide (NaOH, Merck) and 1 M sodium chloride (NaCl, POCH) for 30 min at 4 °C. The preparations were washed three times in distilled water and then incubated in 1 M Tris-HCl (VWR) for 30 min. Then, the slides were dehydrated in an alcohol series: 70%, 90% and 100%. Before applying the anti-5 mC antibodies, the slides were blocked with 5% BSA (Sigma) at RT in a moist chamber for 1h. Immunostaining with the primary antibodies, anti-**5**-methylcytosine antibodies, was performed overnight in a moist chamber at 4 °C. The slides were washed three times in 1 × PBS, after which the secondary antibodies goat anti-mouse IgG antibodies were applied and then incubated in a moist chamber for 1 h at 37 °C. After that, the slides were washed three times in 1 × PBS. The preparations were mounted and counterstained in Vectashield (Vector Laboratories, Peterborough, UK) that contained 2.5 µg/mL DAPI (Serva).

### 4.3. Image Acquisition, Data Analysis and Statistics

The slides were analyzed using a Carl Zeiss Imager Z2 fluorescence microscope with fluorescent lighting H × P, 120W. The images were recorded with an AxioCam ICc5 digital camera and immersions lens with a ×100 magnification.

The frequencies of root cells with micronuclei were analyzed for the control (untreated) and the MH- and MNU-treated meristems. Based on the analyses of the presence or absence of methylated DNA in the micronuclei and their parental nuclei, the frequency of cells with the 5 mC signals was calculated. Additionally, the average fluorescence intensity of Alexa Fluor 488 was analyzed in order to estimate the global level of DNA methylation in the nuclei that were the parental nuclei for the micronuclei and nuclei that did not form micronuclei. The fluorescence intensity of Alexa 488 was measured as the mean values from the integrated density (IntDen) parameter per nuclei using ImageJ (National Institutes of Health; http://imagej/nig.gov (accessed on 18 April 2021)). The integrated density parameter is the sum of all of the pixels within the region of interest (ROI). The eight-bit images with Alexa 488 fluorescence were segmented with the threshold value parameter, and their fluorescence intensities were measured as the mean values from the integrated density parameter. The results of these measurements were estimated in relative units.

The mutagenic treatment experiments using MH and MNU were repeated twice. Three slides, each made from one meristem, were analyzed for each of the two mutagenic treatment experiments. The frequencies of the cells with micronuclei were estimated for 3000 cells for each treatment (control, MH, MNU). Two hundred nuclei per each slide were analyzed in order to estimate the average fluorescence intensity. About 100 micronuclei for each treatment group were analyzed for the presence of the 5 mC signal and average fluorescence intensity. An ANOVA test followed by Tukey HSD test, *p* < 0.05; mean ± SD, were used for statistics.

## 5. Conclusions

In this study, we presented the analyses of DNA methylation in *B. distachyon* nuclei and micronuclei after chemical mutagenic treatments. This is the first example of such a study in plants. To conclude, our results indicated that (1) the level of DNA methylation in mutagen-treated nuclei (both with and without micronuclei) is different if compared to a control and (2) the level of DNA methylation in mutagen-treated nuclei depends on the mutagen type. Our results indicate also that MH and MNU induce micronuclei with different 5 mC levels. The open question remains as to whether DNA methylation has an active role in micronuclei formation, or it is only a marker for chromatin exclusion into micronuclei. More data on the relationship between micronuclei and DNA methylation would be provided using a single-cell genome-wide bisulfite sequencing approach. However, this is technically unfeasible in single Brachypodium nuclei and micronuclei due to their extremely small size. The data on the response of plant genomes to other mutagens that are characterized by different mechanisms of action would also provide more confident conclusions.

## Data Availability

Not applicable.
